# Supplementary figures and images for: Functional analysis of ADARs in planarians supports a bilaterian ancestral role in suppressing double-stranded RNA-response
Source: PLoS Pathog. 2022 Jan 18;18(1):e1010250. doi: 10.1371/journal.ppat.1010250 (PMC8797187; doi:10.1371/journal.ppat.1010250)

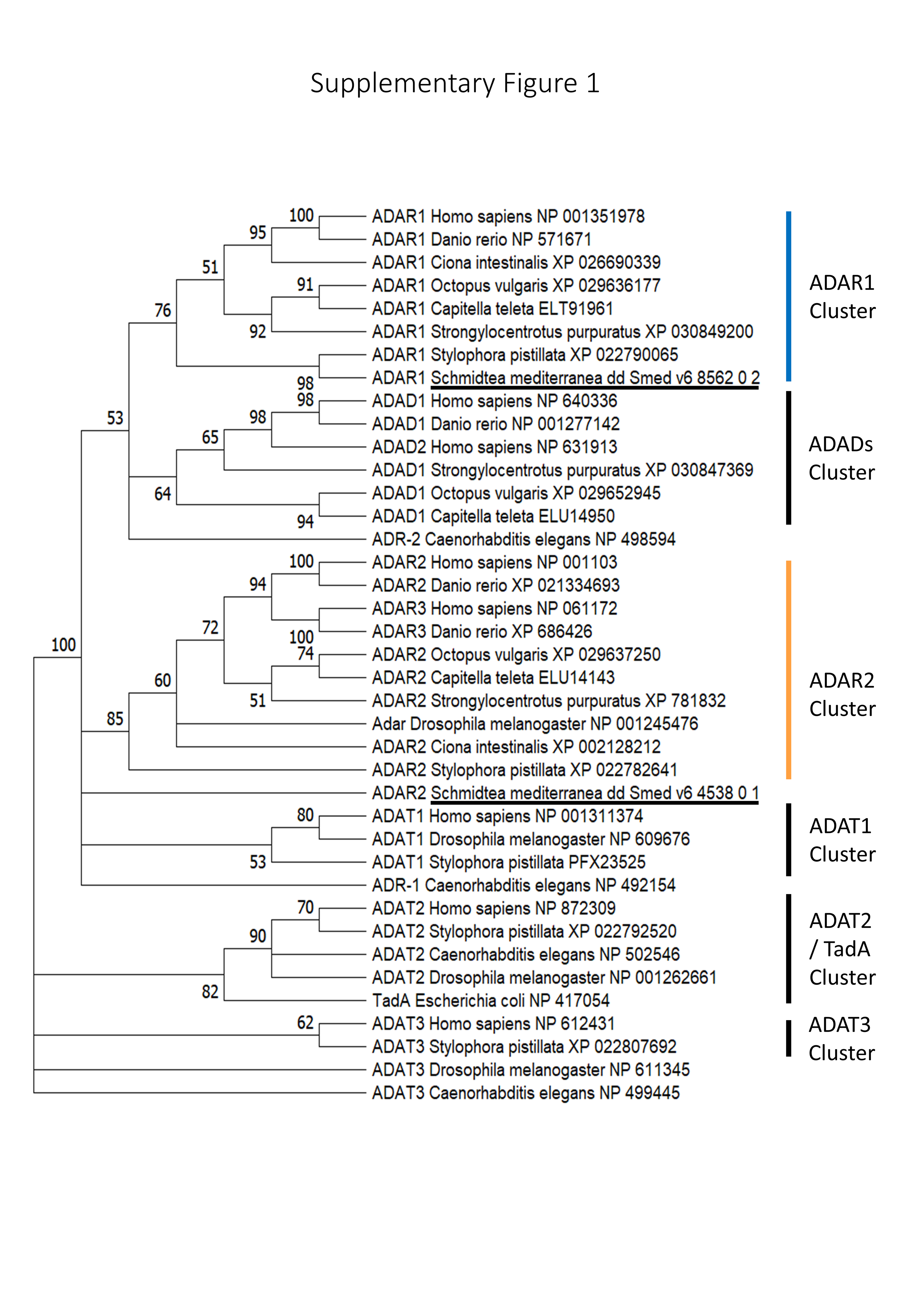

Supplement: S1 Fig — A maximum-likelihood phylogenetic tree of ADAR, ADAD (adenosine deaminase containing domain, also known as TENR), and ADAT (adenosine deaminase acting on tRNAs) homologs, with species representing different bilaterian lineages and cnidarians, places planarian ADAR1 together with its canonical homologs while revealing a high level of divergence in planarian ADAR2. Bootstrap values (percentages based on 1000 replicates) are indicated at the base of the branches. Branches corresponding to partitions reproduced in less than 50% bootstrap replicates are collapsed. (TIF) [file ppat.1010250.s001.tif]

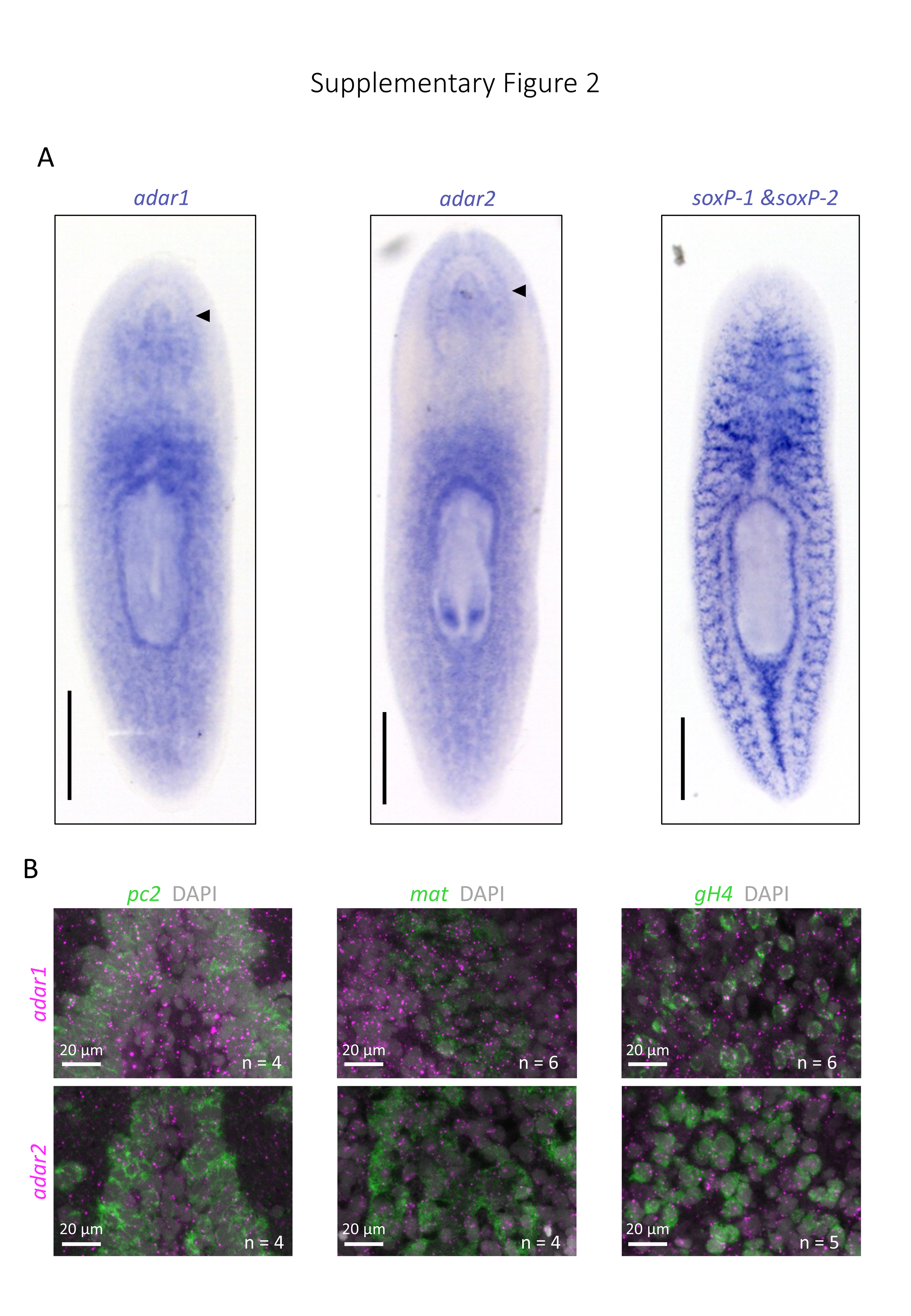

Supplement: S2 Fig — (A) Expression patterns of adar1 and adar2 by WISH (n ≥ 4). Black arrowheads mark enriched expression in the cephalic ganglion. The neoblasts markers soxP-1 and soxP-2 are used to control for probe specificity. Scale bar = 500μm. (B) Representative confocal images of dbFISH show co-expression of adar1 and adar2 (shown in magenta) with neuronal, gut, and neoblast markers (pc2, mat, and gH4, respectively, shown in green). Maximum-intensity projection of a 4 μm section. Scale bar = 20μm. (TIF) [file ppat.1010250.s002.tif]

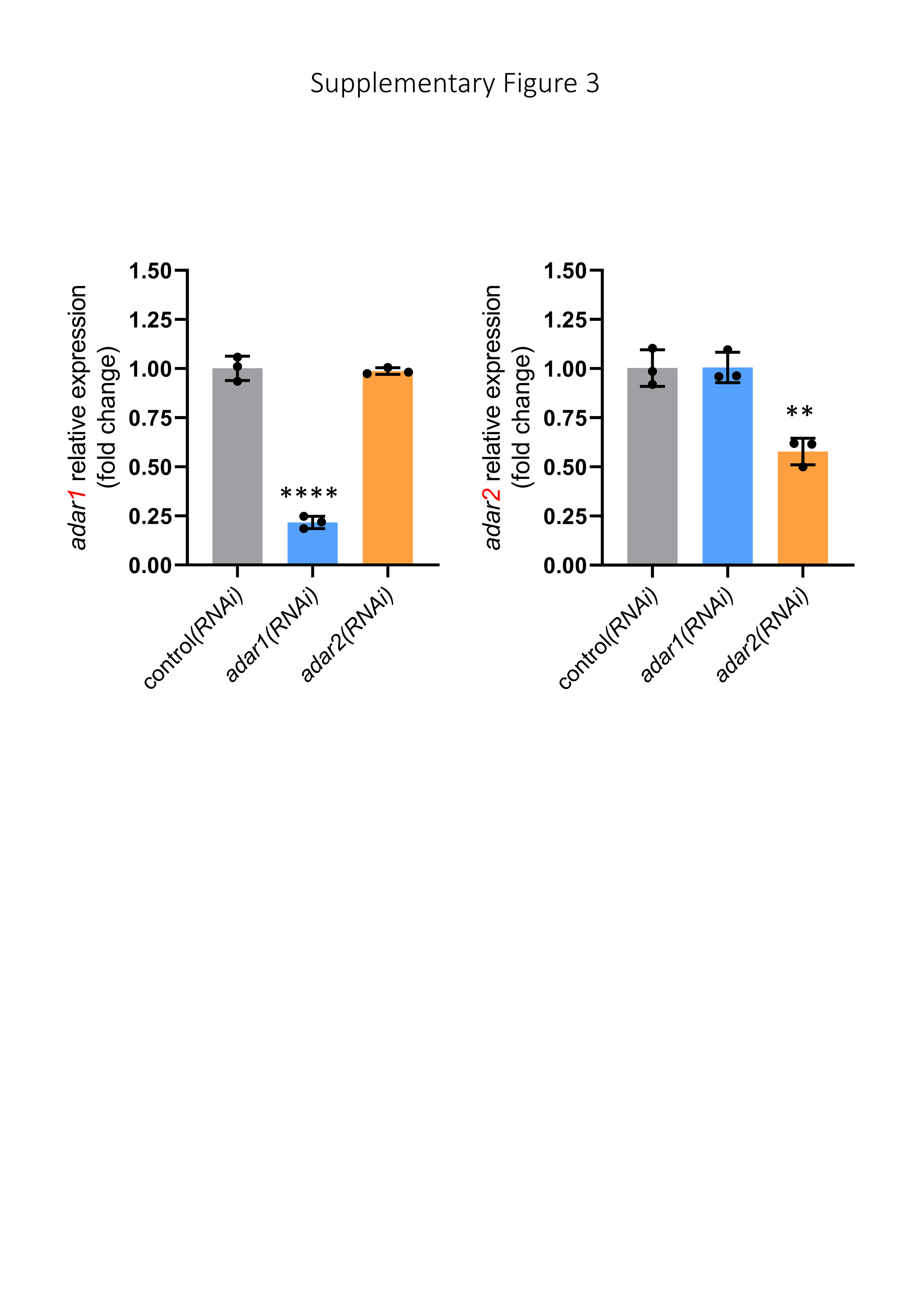

Supplement: S3 Fig — Relative expression levels (qPCR; N = 3 (with three animals that were pooled together in each experiment); mean ± SD) of adar1 (left) and adar2 (right) in adar1(RNAi), adar2(RNAi), and control(RNAi) after 19 days of RNAi. FC = Fold Change. Statistical comparisons are based on one-way ANOVA with Dunnett’s multiple comparisons test (each treatment compared to control). Adjusted p-value ≤ 0.01 (**) and ≤ 0.0001 (****). (TIF) [file ppat.1010250.s003.tif]

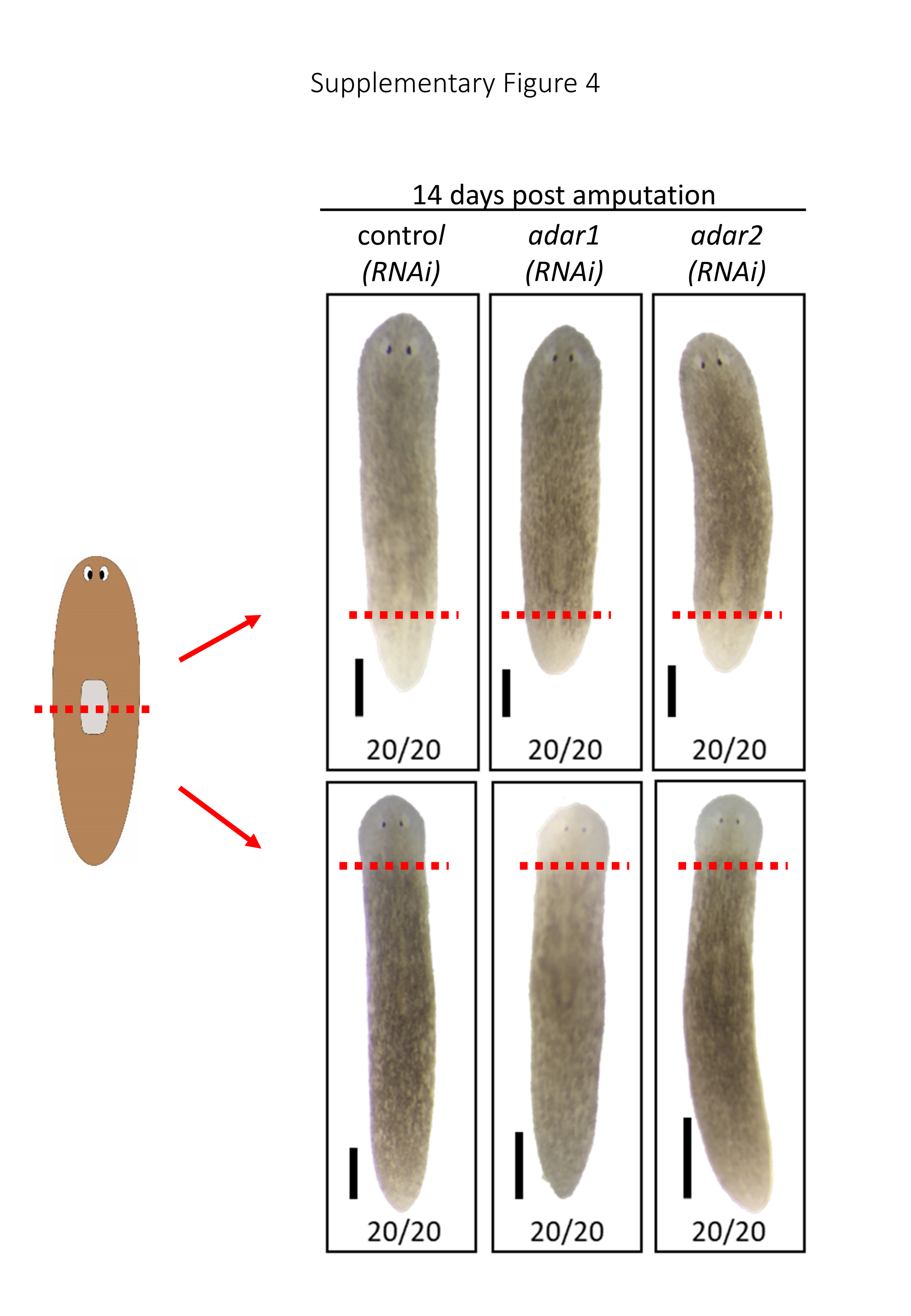

Supplement: S4 Fig — Head and tail regeneration 14 days post-amputation. The red dotted line represents the amputation plane. Worms were fed dsRNA every 4–5 days. n = 20 from two independent experiments (10 worms each) after 19 and 23 days of RNAi (four and five feedings, respectively). Scale bar = 1mm. (TIF) [file ppat.1010250.s004.tif]

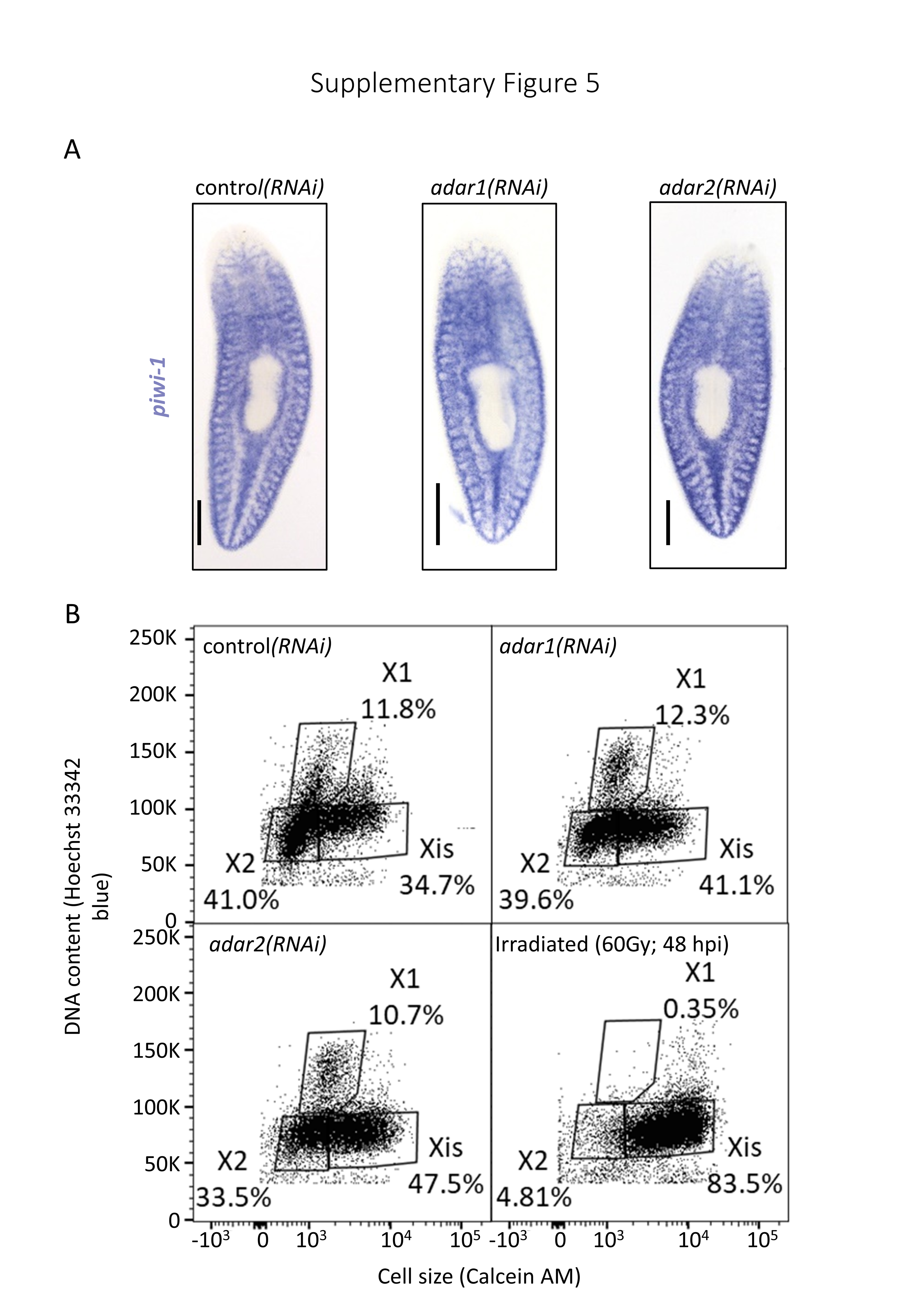

Supplement: S5 Fig — (A) Expression of piwi-1, a pan-neoblast marker, by WISH shows no stem-cell depletion in adar1(RNAi) or adar2(RNAi) animals. Scale bar = 500μm. (B) Cytometry plots quantifying stem cells (neoblasts) (X1 and X2 gates) and post-proliferative cells (Xins) show no stem-cell depletion in adar1(RNAi) and adar2(RNAi) animals after 28 days of RNAi (n = 8). X-irradiated worms served as a positive control for stem-cell loss and gating (60 Gy, 48 hours post-irradiation). (TIF) [file ppat.1010250.s005.tif]

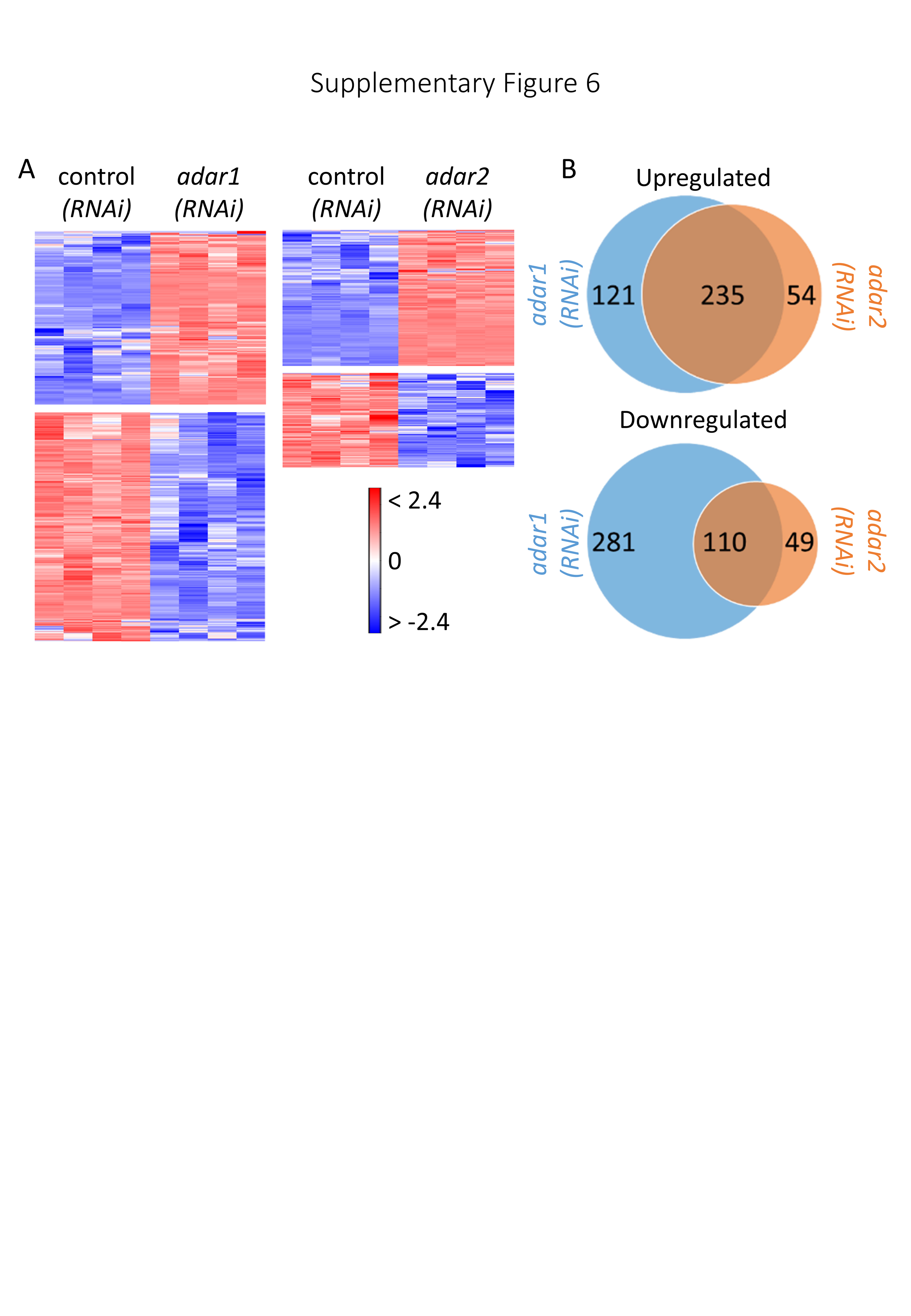

Supplement: S6 Fig — (A) Left—Heat map of 356 upregulated and 391 downregulated genes after 28 days in adar1(RNAi) animals. Right—Heat map of 289 upregulated and 159 downregulated genes after 28 days in adar2(RNAi) animals. N = 4 (with three animals that were pooled together in each experiment); FDR ≤ 0.01; Absolute fold change ≥ 2. The expression values used in the gradient color scheme are normalized log2 CPM values 40. (B) Venn diagram shows an overlap of differentially regulated genes in adar1(RNAi) and adar2(RNAi) animals. (TIF) [file ppat.1010250.s006.tif]

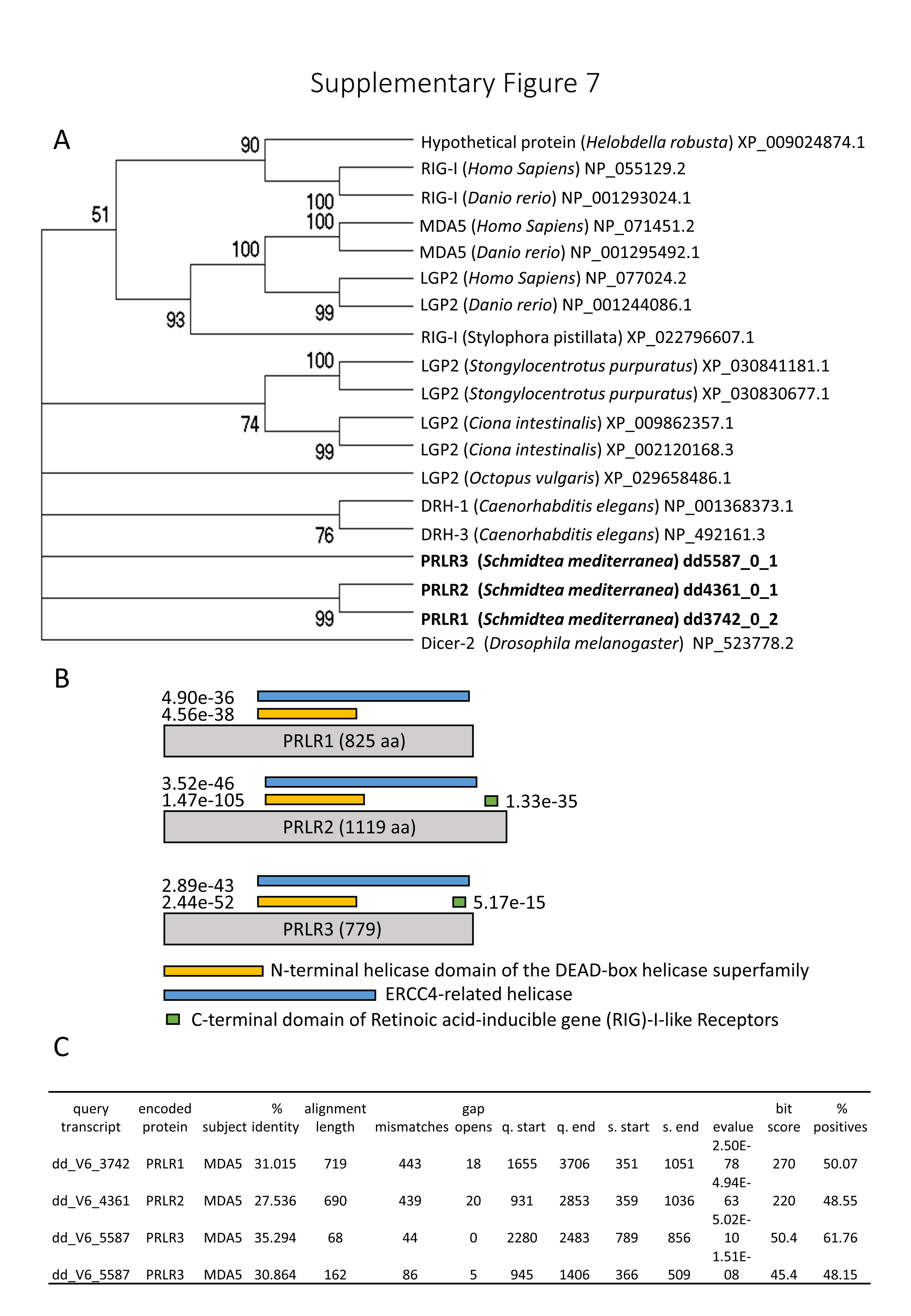

Supplement: S7 Fig — (A) A protein maximum likelihood phylogenetic tree with species representing different bilaterian lineages and cnidarians demonstrates that S. mediterranea RLR homologs are distinct from canonical RIG-I-like receptors (RIG-I, PRLR1, and LGP2). D. melanogaster Dicer-2 served as an outgroup as it harbors a helicase domain homologous to canonical RLRs. Bootstrap values (1000 replicates) are indicated at the base of the branches. Branches corresponding to partitions reproduced in less than 50% bootstrap replicates are collapsed. (B) Domain architecture of PRLR1—PRLR3 as predicted by NCBI conserved domain search 36. E-value scores are indicated next to the identified domains. aa = amino acids. (C) BLASTX analysis of the three planarian transcripts encoding RLR homologs against the protein sequence of MDA5 (human). (TIF) [file ppat.1010250.s007.tif]

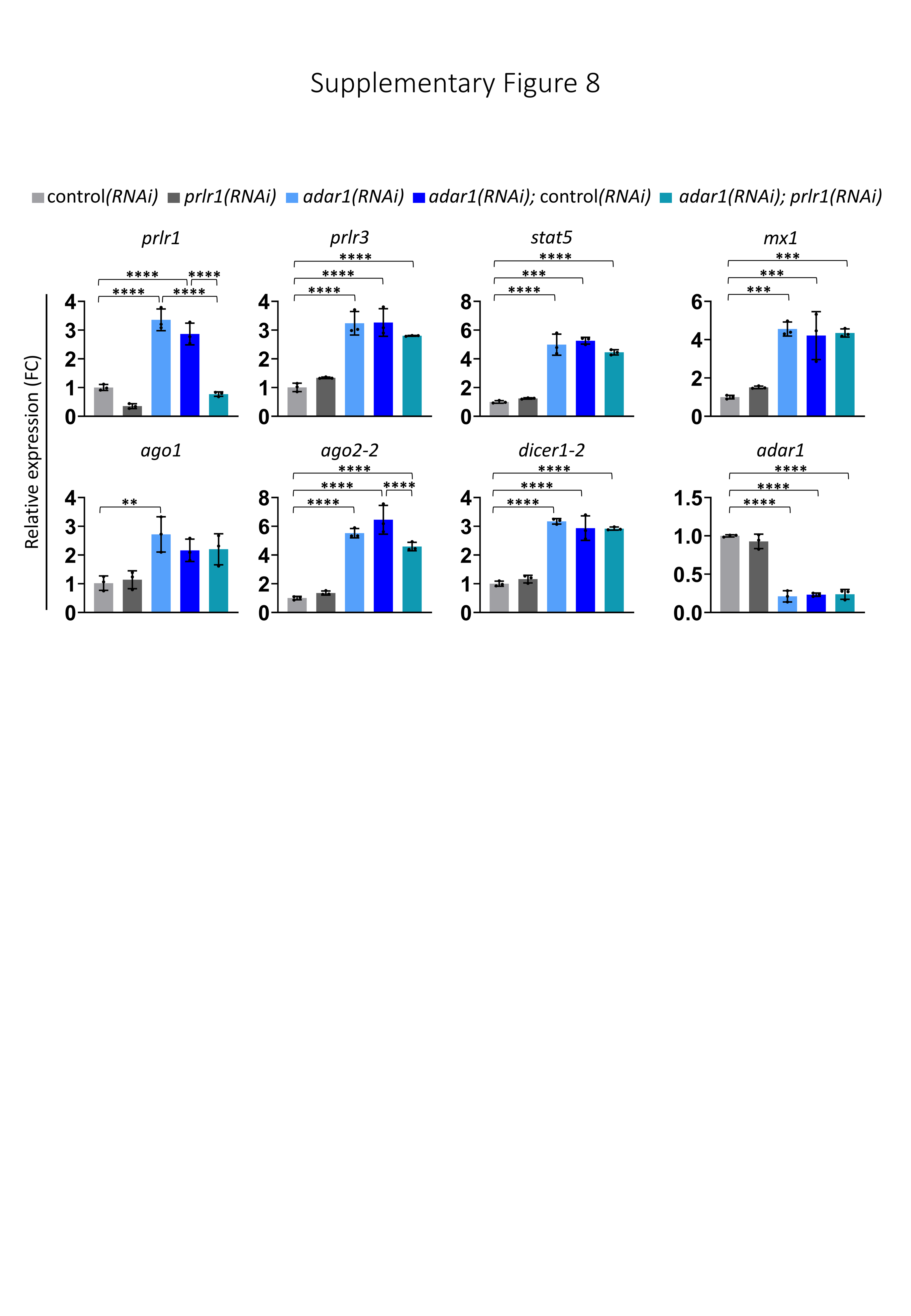

Supplement: S8 Fig — Relative expression levels (qPCR; mean ± SD; N = 3 (with three animals that were pooled together in each experiment)) of seven dsRNA-response genes and adar1 after 14 days of RNAi. FC = Fold change. Statistical analysis—One-way ANOVA with Sidak’s multiple comparisons test. Adjusted p-value ≤ 0.05 (*), ≤ 0.01 (**), ≤ 0.001 (***) and ≤ 0.0001 (****). (TIF) [file ppat.1010250.s008.tif]

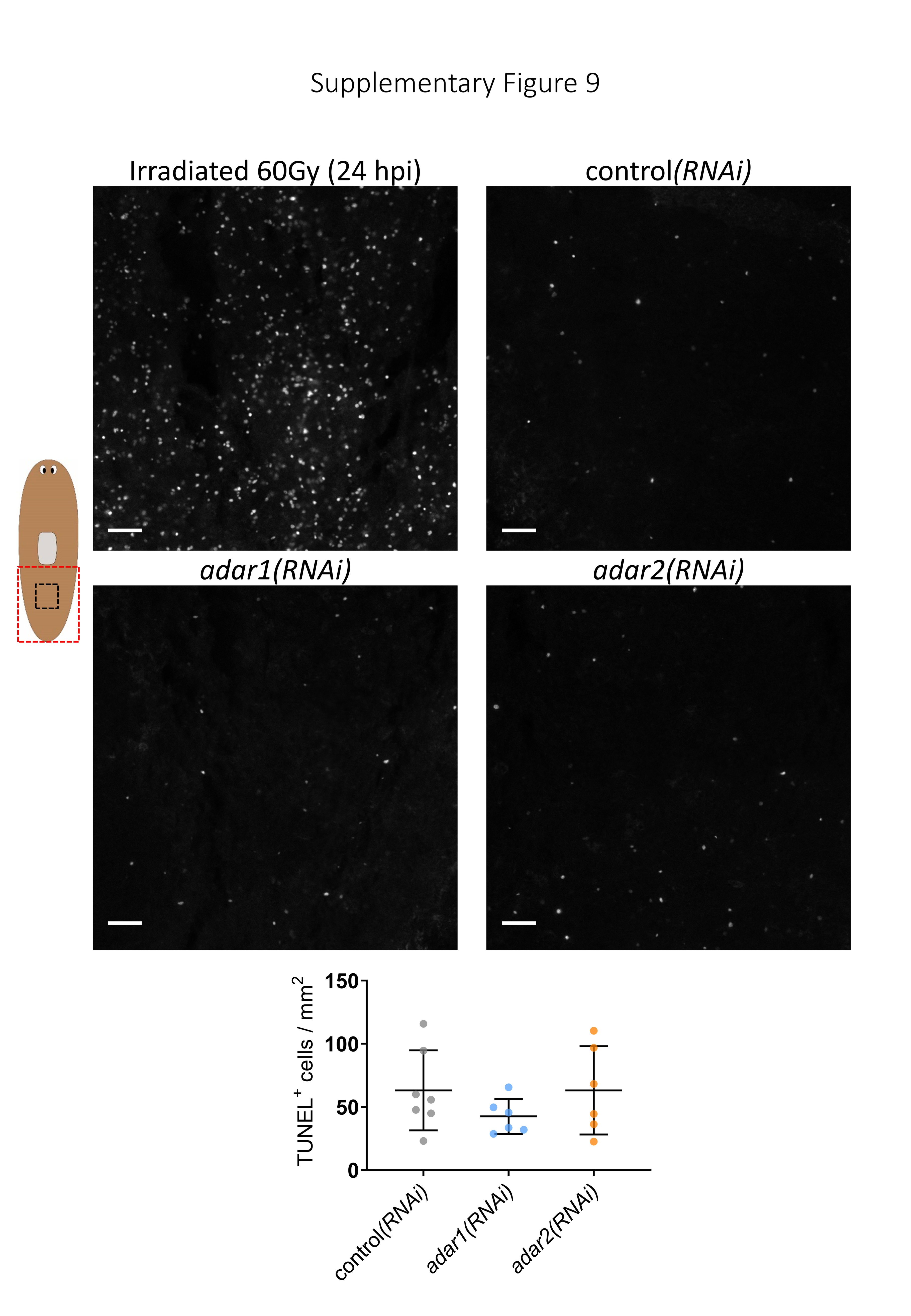

Supplement: S9 Fig — Confocal images (FISH–single plane) and quantification of TUNEL staining after 23 days of adar1 or adar2 RNAi. The dashed black square represents the region corresponding to the images shown in the cartoon, while the red square represents the imaged and quantified area. Scale bar = 50 μm. One-way ANOVA with Dunnett’s multiple comparisons test (each treatment compared to control). No significant differences were detected. (TIF) [file ppat.1010250.s009.tif]

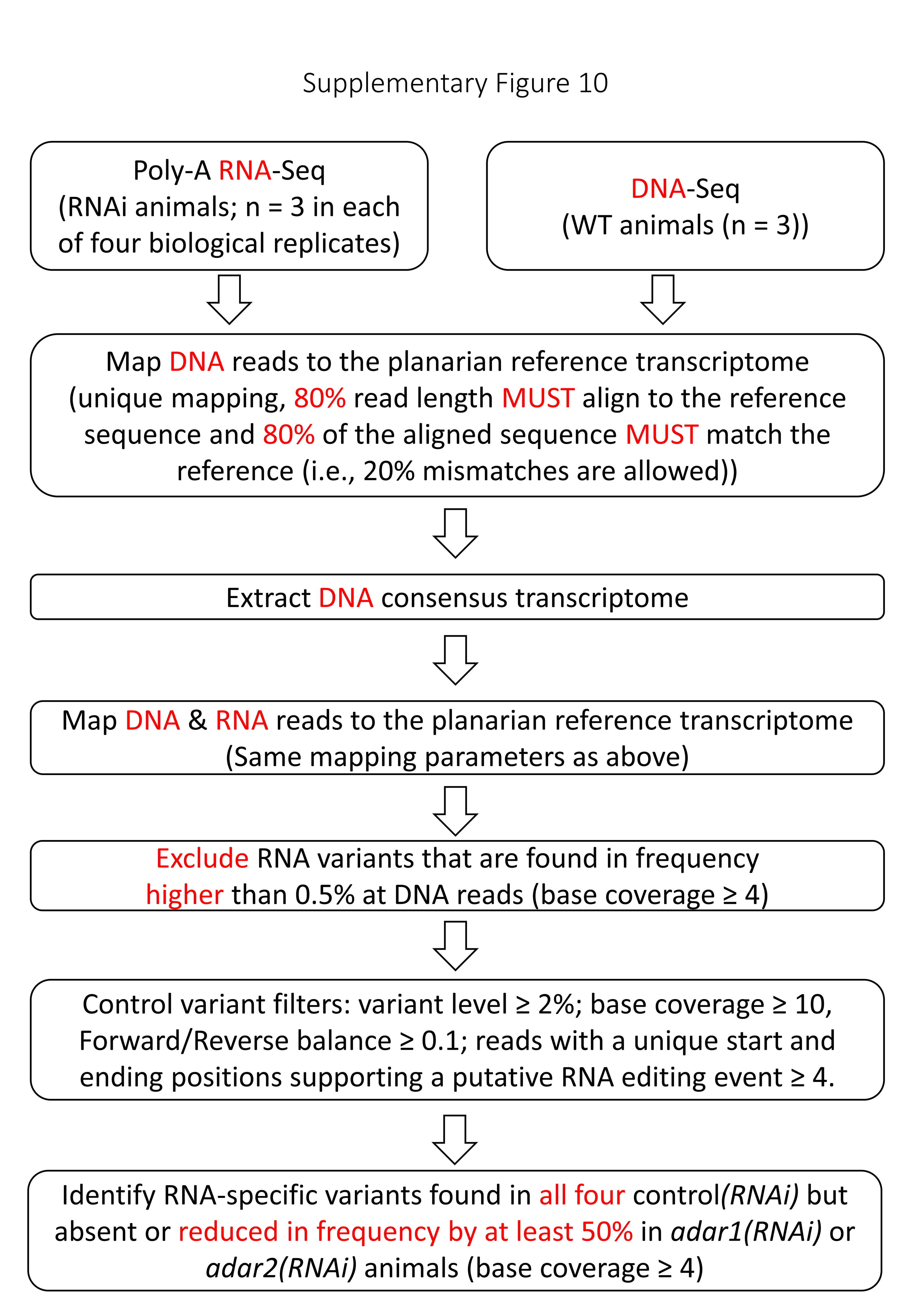

Supplement: S10 Fig — See also the materials and methods section. (TIF) [file ppat.1010250.s010.tif]

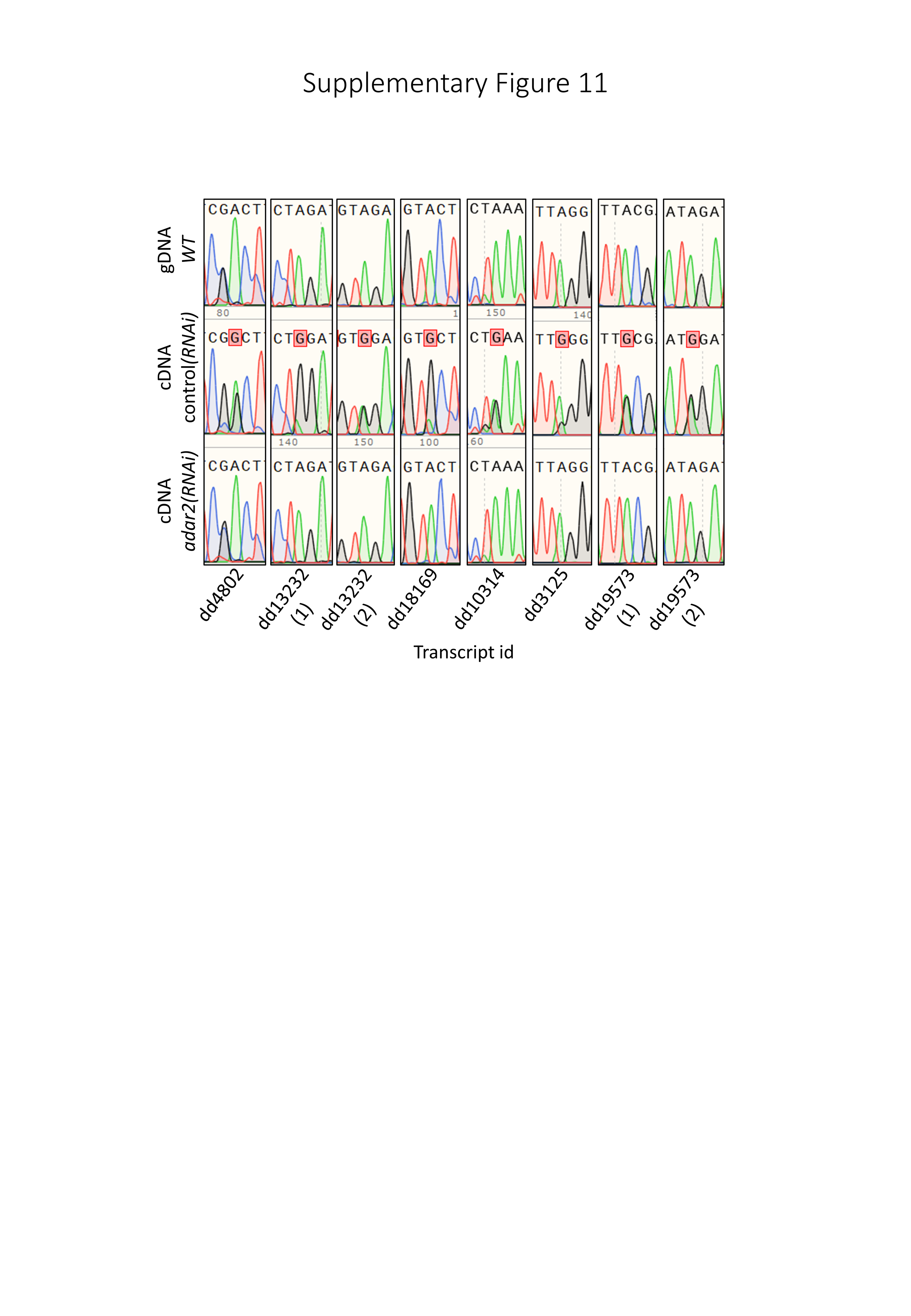

Supplement: S11 Fig — Sanger sequencing validates 8/9 putative A-to-G mismatches identified in our RNA-Seq analysis between adar2(RNAi) and control(RNAi) samples. Here, genomic DNA (WT) and cDNA from adar2(RNAi) animals harbor adenosine in these sites, while cDNA from control(RNAi) animals contain guanosine or mixed guanosine and adenosine (indicative of A-to-I editing). Red boxes denote the validated sites. (TIF) [file ppat.1010250.s011.tif]
