# Supplementary material for: Functional analysis of ADARs in planarians supports a bilaterian ancestral role in suppressing double-stranded RNA-response
Source: PLoS Pathog. 2022 Jan 18;18(1):e1010250. doi: 10.1371/journal.ppat.1010250 (PMC8797187; doi:10.1371/journal.ppat.1010250)
Supplement: Attachment — Submitted filename: Bar-Yaacov_2022_Rebuttal letter.docx [file ppat.1010250.s018.docx]

Thank you very much for submitting your manuscript "Functional Analysis of ADARs in Planarians Supports a Bilaterian Ancestral Role in Suppressing Double-Stranded RNA-Response" for consideration at PLOS Pathogens. As with all papers reviewed by the journal, your manuscript was reviewed by members of the editorial board and by several independent reviewers. The reviewers appreciated the attention to an important topic. Based on the reviews, we are likely to accept this manuscript for publication, providing that you modify the manuscript according to the review recommendations.

Three reviewers found your manuscript interesting and the study well executed. Reviewers 2 and 3 raised a question about phenotype of the double-knockout ADAR1/ADAR2 mutant. Furthermore, the Reviewer 2 asked about a possible time dependence of the observed contrasting knockout effects of respectively ADAR1 and ADAR2 in readout assays used in the study as well as possible roles of PRLR2 and PRLR3 in the ADAR1/2 pathways. Three reviewers also identified relatively minor issues requiring your attention. Finally, any reasonable extension of the virology-related part would be appreciated by the readership of PLoS Pathogens.

Please prepare and submit your revised manuscript within 30 days. If you anticipate any delay, please let us know the expected resubmission date by replying to this email.

Alexander E. Gorbalenya, PhD, DSci

Associate Editor

PLOS Pathogens

Mark Heise

Section Editor

PLOS Pathogens

Kasturi Haldar

Editor-in-Chief

PLOS Pathogens

[orcid.org/0000-0001-5065-158X](http://orcid.org/0000-0001-5065-158X)

Michael Malim

Editor-in-Chief

PLOS Pathogens

[orcid.org/0000-0002-7699-2064](http://orcid.org/0000-0002-7699-2064)

***********************

Three reviewers found your manuscript interesting and the study well executed. Reviewers 2 and 3 raised a question about phenotype of the double-knockout ADAR1/ADAR2 mutant. Furthermore, the Reviewer 2 asked about a possible time dependence of the observed contrasting knockout effects of respectively ADAR1 and ADAR2 in readout assays used in the study as well as possible roles of PRLR2 and PRLR3 in the ADAR1/2 pathways. Three reviewers also identified relatively minor issues requiring your attention. Finally, any reasonable extension of the virology-related part would be appreciated by the readership of PLoS Pathogens.

Reviewer Comments (if any, and for reference):

Reviewer's Responses to Questions

**Part I - Summary**
Please use this section to discuss strengths/weaknesses of study, novelty/significance, general execution and scholarship.

Reviewer #1: The manuscript “ Functional Analysis of ADARs in Planarians Supports a Bilaterian Ancestral Role in Suppressing Double Stranded RNA-Response” by Dan Bar-Yaacov is the first, as far as a I know, functional study on the ancestral role of ADAR activity, revealing that across Bilaterian, it is a key player in reducing the antiviral activity against endogenous viral-like molecules. The author use of Planaria as a model organism to study ADAR activity is novel and unique and thus the author had to establish the system from scratch. The functional experiments are convincing and the paper is nicely written in a clear manner.

Reviewer #2: In this manuscript, Dr. Yaakov identifies two adenosine deaminases acting on RNA (ADARs) of the planarian species Schmidtea mediterranea, an invertebrate belonging to the superphylum Spiralia. S. mediterranea possesses two enzymes ADAR1 and ADAR2, which are evolutionary related to the mammalian ADAR1 and ADAR2 enzymes. This is in contrast to invertebrates of the superphylum Ecdysozoa (including C. elegans and D. melanogaster), which only express a single ADAR more closely related to mammalian ADAR2. In addition, the author identifies three planarian RIG-I-like receptor homologs (PRLR1-3), which are involved in sensing of double-stranded RNA (dsRNA) and induce innate immune responses. Using RNA-interference, the author knocks down expression of ADAR1 or ADAR2 in S. mediterranea and shows that both ADAR1 and ADAR2 knockdown induce expression of a set of genes homologous to mammalian innate immunity genes. However, only knockdown of ADAR1 induces a lethal phenotype in S. mediterranea characterized by increased cell death in the organism. Importantly, the lethal phenotype can be rescued by additional knockdown of PRLR1, indicating a conserved interplay between ADARs and PRLR-signaling as seen in mammals between ADAR1 and MDA-5. Moreover, the increased expression of innate immunity genes upon knockdown of ADAR1 led to reduced replication of a dsRNA virus infecting, S. mediterranea tricladivirus (SmedTV). Finally, Dr. Yaakov shows that ADAR2, but not ADAR1, edits S. mediterranea mRNAs.

The data presented are generally convincing and novel. The identification of a lower organism that has an antiviral innate immune system reminiscent of that in mammals, as well as two ADAR enzymes with seemingly different functions, is very intriguing and may allow further studies to understand the complex interactions of ADARs with the innate immune system in this simple organism. However, a few things are puzzling and should be addressed by the author.

Reviewer #3: The manuscript „Functional Analysis of ADARs in Planarians Supports a Bilaterian Ancestral Role in Suppressing double-Stranded RNA-Response” by Dan Bar Yaacov is the first study of Adenosine Deaminase Acting on RNA (ADAR) proteins in planarian flatworms. Via thoroughly designed and carefully executed experiments, the author shows that S. mediterranea ADARs function as suppressors of a dsRNA / antiviral response and engage in editing of endogenous mRNAs.

Specifically, the author shows via RNAi-mediated knock-downs that ADAR1 is essential for survival of S. mediterranea, but not ADAR2. RNAseq analysis of adar1(RNAi) and adar2RNAi) worms reveals that both regulate the expression of many genes related to viral defence and the response to dsRNA, with a high degree of overlap in the genes suppressed by ADAR1 and ADAR2 (upregulated upon RNAi). The functional significance of the dsRNA response upregulation upon ADAR(RNAi) is demonstrated by the downregulation of the recently described dsRNA virus SmedTV in ADAR(RNAi) worms. Further, the rescue of ADAR1(RNAi) lethality via the concomitant knock-down of a planarian RIG-I-like receptor (prlr1) that the authors identify confirms the deep evolutionary conservation between the ADAR and Rig-like receptors in the basal suppression of the dsRNA response. Finally, the author demonstrates endogenous mRNAs editing by ADAR2, but Interestingly, not by ADAR1.

The manuscript is well written, the experiments are generally of high quality and the discussion doesn't make any undue claims. Overall, the study provides a significant advance in understanding the response mechanisms to viruses and the role of ADARs in recognition of self vs. non-self dsRNA in planaria. Little is currently known about planarian innate immune pathways and viral defence mechanisms, hence the manuscript provides an important foundation for further advances in this research area. In addition, the corroboration of the deep conservation of ADAR functions should be of interest beyond the immediate confines of the planarian research field. Therefore, I consider this manuscript principally suitable for publication in PLOS PATHOGENS.

> We thank the reviews for their kind words and appreciation of our work. Please see below our point-by-point reply.

**Part II – Major Issues: Key Experiments Required for Acceptance**
Please use this section to detail the key new experiments or modifications of existing experiments that should be absolutely required to validate study conclusions.

Generally, there should be no more than 3 such required experiments or major modifications for a "Major Revision" recommendation. If more than 3 experiments are necessary to validate the study conclusions, then you are encouraged to recommend "Reject".

Reviewer #1: (No Response)

Reviewer #2: 1. The author shows that ADAR1 protects S. mediterranea from "autoimmunity" and is proviral for SmedTV, but it does not seem to have editing activity. In contrast, ADAR2 has editing activity in S. mediterranea transcripts, its knockdown reduces SmedTV replication, but seems not to be associated with protection from autoimmune response (lesions and cell lysis), although it also increases expression of innate immunity genes. However, the knockdown efficiency of ADAR1 is much higher than that of ADAR2 (22% vs. ~50% residual transcript levels). The author should investigate, whether the phenotypes are more comparable in a setting where the knockdowns of ADAR1 and ADAR2 are similarly efficient. Maybe with less efficient knockdown of ADAR1, also the lethal phenotype is reduced. In addition it would be interesting to know whether ADAR2 knockdown, which leads to upregulation of innate immunity genes at a later time point than ADAR1 knockdown, will also induce a lethal phenotype with a time delay in S. mediterranea. Is it possible to observe the organisms for a longer period of time? Finally, what happens in a double-knockdown situation of ADAR1 and ADAR2?

> We thank the reviewer for these comments.

Firstly, the reviewer is correct that the knockdown efficacy is higher in *adar1(RNAi)*. However, we wish to stress that after 19 and 28 days (Figure 2), the dsRNA response is comparable between *adar1(RNAi)* and *adar2(RNAi)* animals. Thus, functionally, *adar2* knockdown is sufficient to get the same dsRNA response as *adar1* knockdown (after 19 days).

Second, we were able to knockdown the expression of *adar2* to 41% of its levels in control*(RNAi)* animals, and did not observe any phenotype even in this low endpoint.

Third, knocking down *adar2* was sufficient to eliminate hundreds of putative editing events, thus attesting the loss of the enzymatic activity of ADAR2.

To better communicate this point, we revised the text as following in the discussion (underlined):

Lines 386 – 400: “If the dsRNA-response is harmful, why do *adar1(RNAi)* but not *adar2(RNAi)* animals develop lesions and die? One explanation is that *adar2* was not knocked down sufficiently (Fig. S3). However, three key findings does not support this explanation: 1. The expression levels of dsRNA-response genes were comparable after 19 days of RNAi for both *adars* (Figure 2); 2. Our RNA-seq data that is derived from four independent experiments, and is more accurate than our qPCR analysis, detected that the expression of *adar2* was reduced to ~40% of its levels in control(*RNAi*) animals, yet no phenotype was observed in any of these biological replicates (Table S2); 3. knocking down *adar2* was sufficient to eliminate hundreds of putative editing events (Figure 6), thus attesting to the loss of the enzymatic activity of ADAR2, which indicate that the knockdown was effective. An alternative explanation could be that the rapid induction of the dsRNA-response may be sufficient to induce lesion formation and lysis in *adar1(RNAi)* animals. In addition, *adar1* knockdown has a more significant effect on gene expression than *adar2* knockdown (i.e., the expression of more genes is affected; Fig. S6 and Table S2). Therefore, it is plausible that ADAR1 has additional roles impaired by knockdown when combined with the induction of the dsRNA-response apply cumulative stress that leads to lethality in *adar1(RNAi)* animals.”

> We also agree that it is important to examine the appearance of lethal phenotype in a “later time point”. This is precisely why we continued our experiments and knockdown *adar2* for more than two months (Figure 1). Notably, This time frame is three times longer than the time it took *adar1(RNAi)* animals to start having lesions. Moreover, not even a single *adar2(RNAi)* animal died or displayed a phenotype even after 65 days. As mentioned above, when it would be possible to knockout genes in planarians and abolish expression altogether, then we will be able to ask if ADAR2 is also essential in planarians.

> We agree that examining the interaction of ADAR1 with ADAR2, other PRLRs, and other genes is interesting. However, these experiments are a part of a follow-up project we are now designing, and are beyond the scope of this manuscript that aim to examine the link between ADARs and the innate antiviral dsRNA response.

2. PRLR1 rescues the lethal phenotype of ADAR1 knockdown. However, the author has identified three PRLRs. He should also test whether PRLR2 and PRLR3 are also involved in the autoimmune response in S. mediterranea after ADAR1 knockdown. At least from the homology analysis (Fig. S7) it appears as if they have the C-terminal domain known to be important for RIG-I function in mammals, and thus might be relevant. In addition and to strengthen the virology aspect of the study, it would be interesting to establish a role for the PRLRs in infection with SmedTV.

> We thank the reviewer for this suggestion. Indeed, as mentioned in our previous comment, we plan to examine the interaction of ADAR1 with each of the PRLR genes in the framework of a large follow-up project. Indeed, PRLR1 rescued the phenotype caused by *adar1* RNAi. At this time point the project required prioritization, which led to our focus on PRLR1. In the future we will also examine the effect of each of the PRLRs (that affect the dsRNA response) on SmedTV infection, yet currently it is outside the current scope of our manuscript.

3. ADAR-editing in S. mediterranea transcripts seems to be dependent on ADAR2, whereas ADAR1 seems not to have editing activity despite having a putatively functional deaminase domain. The author should check whether editing occurs in SmedTV RNA and, if so, whether ADAR1 and/or ADAR2 are involved.

> We agree with the reviewer for this excellent suggestion. To address it we performed the suggested analysis and did not observe any editing events in the RNA of *SmedTV*. TO communicate this finding, we added the following sentences to the results and the discussion sections:

Results, line 348-350: “Notably, ADAR1- or ADAR2-dependent putative mRNA editing events were not found in SmedTV’s RNA. Thus, the observed effect of adars(RNAi) on SmedTV RNA and infected cells, is likely editing-independent.”

Discussion, lines 409-411: “Finally, we could not detect putative mRNA editing events in the RNA of SmedTV. Thus, the effect of ADARs on the abundance of *SmedTV*’s RNA and infected cells is likely indirect.”

Reviewer #3: One of the important unresolved issues is the substantial overlap in genes suppressed by ADAR1 and ADAR2, yet the apparent disparity in lethality and RNAi editing capacity of the two homologues. Double knock-downs of the two ADAR homologues would seem to offer a straight-forward and potentially informative approach to further test the extent of functional redundancy between the two homologues, especially with respect to the rate of onset and end point of the effect (e.g. SmedTV downregulation). In case additional experiments are not feasible, the respective section of the discussion should go into more detail and integrate knowledge from other model organisms on possible redundant roles of ADARs.

> We thank the reviewer for this suggestion. Please see our reply to reviewer #2. As suggested, we expanded the respective section in the Discussion, as following:

Discussion section, lines 412-420: “In mice, ADAR1 is responsible for most editing events, especially in non-coding regions, while ADAR2 mainly edits coding regions ^2^. Knocking out each Adar gene individually leads to a lethal phenotype ^1,11-13^. Thus, at least in part, the activities of ADAR1 and ADAR2 are not redundant. Since *D*. *melanogaster* and *C. elegans* harbor only a single ADAR gene ^21,45^, investigating the redundancy between ADAR homologs in these model systems is impractical. Planarians, however, harbor two ADAR homologs. Our results indicate both similarities and differences upon *adar1* and *adar2* knockdown in planaria. Thus, planarians could serve as an attractive model for investigating the interaction between ADAR paralogues. Finally, we and others ^19,22^ showed that ADAR2 orthologue could induce a dsRNA response phenotype, it may be of interest to examine its involvement in antiviral dsRNA response also in mammals.”

**Part III – Minor Issues: Editorial and Data Presentation Modifications**
Please use this section for editorial suggestions as well as relatively minor modifications of existing data that would enhance clarity.

Reviewer #1: I had the opportunity to review an earlier version of this paper elsewhere before- the current version is well improved and much of my earlier comments were resolved.
Currently I have only few minor comments (none is mandatory) :
1- It can be nice to add more analysis to the RNA editing section of the paper:
a. Why so few editing sites were found (although the signal is very clear) is it possible that the cutoff were too stringent?

> We thank the reviewer for this point.

First, we wish to emphasize that the cutoffs already allow detection of hyper-editing events as we allowed read mapping with 20% mismatches against the reference sequence.

Second, we accept the reviewer suggestion and repeated the analysis and allowed detection of editing events with a coverage of 10 reads (instead of 20). The latter was the only altered parameter to avoid increasing the risk for detecting false positive editing sites. Moreover, we have further improved our detection pipeline to better exclude false positive sites. Now we only apply our stringent filters on our control samples. Specifically, we allowed variant detection in our RNAi and DNA samples on sites that are covered with a minimum coverage of four reads. Also, we remove sites that have a variant in a frequency greater than 0.5% in the RNAi or DNA samples. These new criterions, allow the elimination of false positive sites that are found either in the RNAi treated or DNA samples.

We changed the text in the Materials and Methods section accordingly.

Lines 532-542: “To identify edited sites in transcripts, we first excluded mismatches between our RNA sequences and the DNA-based consensus transcriptome present in our DNA-Seq reads (sites with a variant frequency above 0.5%). Second, we kept only mismatches with a frequency of at least 2% in the control animals. All identified sites had sequenced-read coverage ≥ 10, with four unique reads supporting putative RNA editing events. Third, only mismatches that were found in all four control*(RNAi)* samples but were absent or reduced in frequency by at least 50% in *adar1(RNAi)* or *adar2(RNAi)* animals were called. Notably, we allowed the detection of editing events in *adar1(RNAi)*, *adar2(RNAi)* and DNA samples with coverage of only four reads, to exclude false positive sites identified due to lack of data in these samples.”

We revised Figure S10 accordingly.

Now, mismatches that are not the result of A-to-I editing (A>G or T>C) only represents 2.5% (6/246) of the sites (>9% before). We revised Figure 6 accordingly.

b. Does editing sites tend to take place within mobile elements in the Planaria genome?

>To address this comment, we re-examined our editing events, and found that only one site occur in a transcript with homology to known transposable elements (Table S4). To communicate this point we added the following text in the Results section.”

Lines 343-346: “In humans, editing events tend to occur in inverted Alu repeats ^43^. In contrast, except for one site, none of the identified putative edited sites occur in transcripts with homology to known transposable elements. However, the planarian genome is still far from fully annotated, so we cannot exclude editing events in additional planarian-specific transposable elements.”

c. Does editing sites tend to take place within predicted dsRNA structures?

>We thank the referee for this excellent suggestion. We performed the relevant analysis and added text to communicate the results of this analysis:

Line 351-356: “ADARs are known to edit dsRNA structures ^2^. Therefore, we analyzed all putative 240 A>G sites, using RNAfold, to detect dsRNA structures across all identified edited transcripts. Our analysis revealed 171 sites that are predicted to pair with a different site in the transcript (Table S4). Of these, 106 sites are predicted to be embedded in a dsRNA stretch larger than three bases. Since the planarian homolog of ADAR2 contains a dsRNA binding domain (Figure. 1A), it is plausible that it also targets dsRNA structures, similar to ADAR proteins in other organisms.”

We also added the following text to the Materials and Methods section:

Line 545-550: “**RNA secondary structure prediction**

In order to examine the RNA secondary structure, we used a locally installed RNAfold program (RNAfold ViennaRNA-2.5.0) to predict the RNA secondary structure with minimum

free energy. We analyzed the entire set of edited transcripts, and examined whether edited sites could pair and what is the length of a detected dsRNA structure, providing that at least 80% of the bases are paired.“

d. Any correlation between editing level across tissues and ADARs expression levels?

>We performed this analysis and found no such correlation . This is most probably because the single-cell RNA-seq (scRNA-seq) database we used did not detect any expression enrichment of either *adar1* or *adar2* in specific tissues. However, we suspect that no enrichment was observed because scRNA-seq only captures the most abundant transcripts (not *adars*). In the future, it would be interesting to revisit this analysis when the sensitivity of scRNA-seq techniques increase.

Reviewer #2: A few additional minor comments:

4. It is mentioned that the ability of S. mediterranea to regenerate amputated heads or tails was not affected by knockdown of either ADAR1 or ADAR2, when performed before onset of lesion formation (in case of ADAR1 knockdown). Please indicate how much ahead of time these amputation experiments were performed. What happens, when amputation is performed closer to the onset of lesion formation?

>Amputations were performed at 19 and 23 days after RNAi treatment commenced (just before lesions appeared). Amputating the animals when sick or injured due to the RNAi treatment cannot distinguish between the direct effect of *adar1* or *adar2* knockdown. That is because additional processes may occur, such as infection of secondary cellular failure that will damage the regeneration process. It was shown in previous studies that if a gene has a profound role in neoblasts (stem cells) regulation or tissue regeneration, its effect on animals’ regeneration is noticeable before the appearance of any visible phenotypes.

To better communicate the time of amputation relative to lesions formation, we added the following text in the results section (underlined):

Lines 130-131: “performed no more than five days before lesions formed in *adar1(RNAi)* animals.”

5. The tables in the supplementary file S2 would be easier to screen if the author would include an additional column with a common protein or gene abbreviation of the homologs. Some, but not all identified homologs, have abbreviations included in the full names.

> We agree and added the suggested column in Table S2

6. Is it possible to quantify the frequencies of SmedTV-positive cells in figure 3 by flow cytometry? The results may be more accurate than quantification from fluorescent microcopy images.

> We agree it could be helpful, but this experiment is currently not feasible as far as we know.

Reviewer #3: 1) Figure 1B: While the phenotype of adar1(RNAi) worms is clearly visible as described in the text, the images also suggest that adar1(RNAi) animals are much smaller than control- and adar2(RNAi) worms. The adar1(RNAi) worm that is shown for the mild/moderate phenotype has a length of about 6 mm while the control- and adar2(RNAi) worms are about 11 mm in length. Is this difference representative for the whole adar1(RNAi) cohort and maybe due to impaired growth in adar1(RNAi) worms? If this is the case, please report the phenotype in the text. If not, please change the panel accordingly to show a worm of representative size for each cohort.

>We thank the reviewer for noticing this difference. Indeed, all *adar1(RNAi)* animals are smaller than their *control(RNAi)* and *adar2(RNAi)* animals. This is due to the deleterious effect of knocking down *adar1* levels. To communicate the phenotype, we added the following text to the results (underlined here):

Line 125-126: “All *adar1(RNAi)* animals were smaller than control (RNAi) animals, developed lesions, and 73% (44/60) died (Fig. 1B and 1C).”

2) Lines 217-223 & Figure 3A-B: In the main text, figure 3 and figure legend it is not clear whether SmedTV positive cells were only quantified in the head areas shown in Figure 3A or whether cells in the whole body were quantified. Please clarify the analysis. If only SmedTV positive cells in the head areas were quantified, please give a statement or quantification whether a similar effect is visible in other areas of the body. Burrows et al. 2020 showed that SmedTV positive cells are found across the whole body of S. mediterranea. Since the phenotype of ADAR1(RNAi) is least pronounced in the head region (Figure 1B), the effect of ADAR1(RNAi) on SmedTV positive cells might be higher in other regions of the body. Lines 105-106 & Figure S2A: From my point of view, there is no apparent enrichment visible in the brain for WISH stainings of adar1 and adar2. The signal even seems to be weaker in the anterior part and head, respectively, than in the rest of the body. It also seems like there is an enrichment of the signal in cells surrounding the pharynx. Please explain why you conclude an enrichment in the brain or adjust the description of the stainings in the text.

> We thank the reviewer for this important comment. SmedTV was shown to infect neurons (Burrows et al. 2020). In our hands (and animals), the majority of SmedTV’s signal in WT animals following RNA *in situ* hybridization stemmed from the head of the animals and specifically from their cephalic ganglion. Therefore, we focused on the head region of the animals to enable thorough imaging of the most infected tissues. This allowed us to carefully and accurately quantify SmedTV-positive cells. Notably, in both *adar1(RNAi)* and *adar2(RNAi)* animals, the signal is less pronounced than in control(*RNAi)* animals. We interpret this difference as the result of hyper-activated and systemic antiviral activity. To better communicate this analysis, we added the following text to the Results section (added text is underlined):

Line 233-235: “we sampled more than 20 worms (pooled from two independent experiments) and counted the number of infected cells in the head of the animals (Fig. 3A), as we observed that the majority of SmedTV infected cells, across RNAi treatments, resides in the cephalic ganglion of our sampled animals.”

3) Line 110: I think an “of” is missing: “… , we used RNAi knockdown of gene expression.”

> Done.

4) Lines 113-114: It should be clear that the morphology of adar2(RNAi) animals is not different from control/wildtype worms. Please change the wording accordingly.

> We changed the text as suggested (underlined):

Line 127: “…display any gross morphological phenotype changes, and were similar to control*(RNAi)* animals…”

5) Lines 256-264 & Figure 4: Please clarify at which timepoint and after how many RNAi feedings, respectively, the phenotype observations were done. Please also include the more detailed explanation of the feeding schedules (initial feeding of only prlr1 dsRNA or control dsRNA) as written in the methods section lines 442-444, in this results section to make the RNAi treatment immediately clear.

> We changed the text as suggested (underlined):

Lines 273-275: “Moreover, lesions started to appear after only four feedings of dsRNA (19 days) in all *adar1* RNAi treatments (single and double RNAi treatments). However, in *adar1(RNAi); prlr1(RNAi)* animals, their severity and frequency decreased (Fig. 4A-B)”.

Regarding adding the full protocol of the double RNAi treatments, we prefer to keep these detailed and purely technical descriptions in the 'Materials and Methods' section, as we think that it may distract the reader from the findings, and interrupts the flow of the paper. Therefore, we added a reference to the Materials and Methods section.

Line 271: “(see materials and methods).”
